# Reliability of a Qualitative Instrument to Assess High-Risk Mechanisms during a 90° Change of Direction in Female Football Players

**DOI:** 10.3390/ijerph19074143

**Published:** 2022-03-31

**Authors:** Alba Aparicio-Sarmiento, Raquel Hernández-García, Antonio Cejudo, José Manuel Palao, Pilar Sainz de Baranda

**Affiliations:** 1 Research Group “Raquis: Aparato Locomotor y Deporte”, Department of Physical Activity and Sport, Faculty of Sport Sciences, Campus de Excelencia Internacional Mare Nostrum, University of Murcia, 30720 Murcia, Spain; rhernandezgarcia@um.es (R.H.-G.); psainzdebaranda@um.es (P.S.d.B.); 2 Scientific Association of Research Groups “Sport Performance Analysis Association”, 30720 Murcia, Spain; palaoj@uwp.edu; 3 Department of Health, Exercise Science & Sport Management, University of Wisconsin-Parkside, Kenosha, WI 53144, USA

**Keywords:** movement quality, injury risk, soccer, sidestep, cutting task, knee load, ligament injuries

## Abstract

Sidestep cuts between 60° and 180° and one-leg landings have been identified as the main mechanisms of ACL injuries in several sports. This study sought to determine intra- and inter-rater reliability of a qualitative tool to assess high-risk movements in a 90° change of direction when the test is applied in a real framework of sport practice. Female footballers from two teams (*n* = 38) participated in this study and were asked to perform 90° cutting trials to each side, which were simultaneously filmed from a frontal and a sagittal view. A total of 61 cases were selected for 2D qualitative observational analysis by three raters. Poor reliability was found among each pair of raters as well as moderate reliability when the Cutting Movement Assessment Score (CMAS) was given by the same rater at different moments, but with too high a minimum detectable change. On the other hand, raters presented a significant, as well as moderate-to-good intra-rater reliability for most items of the CMAS tool. There was, however, non-significant reliability between observers in rating most check-points of the tool. For these reasons, more objective guidelines and clearer definitions for each criterion within the CMAS, as well as a longer, standardised training period for novel observers, would be highly recommended to improve the reliability of this tool in an applied context with female footballers.

## 1. Introduction

Deceleration manoeuvres such as landing or change of direction (COD) are frequent and potentially high-risk actions in team sports [1,2,3]. Sidestep cuts between 60° and 180° and one-leg landings have been identified as the main mechanisms in ACL injury—inciting events in several sports [4,5,6,7,8,9,10]. In football, most movements are not performed in a forward direction, so COD manoeuvres are especially frequent and relevant in this sport, with CODs up to 90° being the most frequently performed action [1]. A recent investigation with Japanese players suggested the sidestep cut as the most frequent action during which female players can tear their ACL [5]. Lucarno et al. (2021) [11] stated that ACL injuries in professional female footballers predominantly occur without direct contact and during defensive actions, such as pressing and tackling, and with frequent knee valgus loading.

Knee abduction motion, most commonly known as knee valgus, has been identified as a factor strongly related to a higher load on the knee joints and potential ACL loading in the performance of landing and COD mechanics, especially in women’s sports [7,12,13,14,15,16,17]. Some prospective studies have found that the knee abduction moment (KAM) as well as knee medial displacement in landing tasks are key predictors of a higher potential to suffer an ACL injury in female athletes [15,16,17,18].

Alignment (moment arms) while performing a sidestep cutting task has been shown to have a substantial impact on knee valgus moments, even more so than the magnitude of forces [7]. Sigward et al. (2015) [16] carried out a study with footballers (including females) and determined that greater hip internal rotation and knee valgus angles were predictors of larger knee valgus moments during cutting manoeuvres performed between 45° and 110°.

The braking strategy during the penultimate foot contact (PFC) of a COD can also influence knee-joint load during the final contact of a sidestep cut [19]. Jones et al. (2016) [20] carried out a study with 22 female footballers and determined that the penultimate contact plays a role in braking and preparing the body for an optimal position for final contact during COD manoeuvres. A better braking strategy during PFC has also been found to improve the ability to perform a faster COD [21].

Other biomechanical factors—such as limited knee flexion [2,22], insufficient trunk flexion displacement during FFC [3,12,23], non-neutral foot position [24], knee valgus [25,26], lateral trunk flexion towards the stance leg [12,26,27] or a wide lateral leg plant distance [26,28]—have also been found to be key technical determinants of potentially hazardous knee-joint loads during sidestep cutting. In particular, Jones et al. (2015) [26] studied technique determinants of knee-joint loads during cutting in female footballers and found that initial knee abduction angle, lateral leg plant distance and initial lateral trunk lean during a 90° cutting manoeuvre could explain 67% (62% adjusted) of the variation in peak KAMs. Such findings are very relevant as they indicate that there are potential modifiable technical factors to lower peak KAMs during cutting. The biomechanical evaluation of 60°-to-180° sidestep cuttings might thus be relevant to detect female footballers at higher risk of injury as well as to guide the preventive training or the rehabilitation process to reduce knee loads during cutting [26,29,30,31].

The need for sophisticated and expensive laboratory instruments makes the calculation of external KAM less accessible and difficult to apply in field-based contexts by sports and conditioning professionals [28]. For that reason, different field-based tests that evaluate landing mechanics have been developed and have been shown to be valid and reliable in estimating knee load or KAM in female athletes [32,33,34,35,36]. In contrast, few studies have evaluated COD mechanics to analyse the risk of ACL injury in sports, with very few applying valid and reliable field-based procedures and instruments [37,38]. Weir et al. (2019) [39] developed a valid and reliable method for 2D biomechanical analysis of several kinematic variables in an unplanned 45° COD manoeuvre to estimate KAM in female athletes. In addition, Della Villa et al. (2021) [40] developed an assessment protocol based on kinematic measures correlated with higher KAM when footballers performed a 90° sidestep cutting task. These protocols are not very time-efficient, however, and require the participation of sports professionals with a specific qualification in kinematic video analysis, which complicates applications in a real context.

In a recent study, a specific and accessible tool was designed, called the Cutting Movement Assessment Score (CMAS), for the qualitative observation of certain high-risk mechanics in a 90° COD manoeuvre [41]. These authors expanded on their preliminary investigation and explored the validity and reliability of the CMAS tool with 28 males and 13 females from several sports [42]. They confirmed that a higher CMAS score was highly correlated with greater KAM exhibited during the final contact of a 90° COD. Additionally, subjects with a higher CMAS score displayed higher-risk cutting postures, including greater peak knee abduction angles, internal foot progression angles and lateral foot plant distances (*p* ≤ 0.032, effect size = 0.83–1.64). Their results also showed moderate-to-excellent intra- and inter-rater reliability (ICC from 0.690 to 0.946) using the CMAS tool.

However, in the study by Dos’Santos, McBurnie et al. (2019) [42], the COD task was carried out and recorded in a laboratory setup with athletes of both sexes who practised different sports. It therefore remains unknown whether the CMAS tool would be reliable enough when analysing a 90° COD task recorded on both sides in a sport-specific context and performed by a particular sample of athletes. Intersession reliability also has not been previously studied using the CMAS tool. This study therefore sought to determine the intra- and inter-rater reliability using the CMAS tool to assess the movement quality of a 90° COD when the test is applied in the real framework of sport practice with female footballers. A second aim was to ascertain the inter-session reliability to estimate the reproducibility of the protocol with the execution of the test in different sessions. The main results showed a moderate-to-good intra-rater reliability for most items of the CMAS tool, although there was non-significant reliability between observers.

## 2. Methods

### 2.1. Experimental Approach and Design

First, this study had a cross-sectional design to determine the reliability of the CMAS tool to assess 90° cuts to both sides using a specific field-based approach in football. Second, a within-subject, repeated measures, pre-to-post design (test–retest) was used to study the reproducibility of the protocol over two sessions (one week apart). Two-dimensional video footage data were captured to allow for observational qualitative screening using the CMAS. Both testing sessions were carried out in January 2020, and at that time, players were in their competitive phase of the season. The observational analysis was performed from March to April 2020.

### 2.2. Participants

The minimum sample size of 26 was determined based upon the correlation value of r = 0.63 (based on the Spearman’s correlation found between CMAS and KAMs (ρ = 0.633; *p* < 0.001) in the preliminary investigation of Jones et al. (2017) [41]), a power of 0.95 and type 1 error or alpha level of 0.05 calculated using G*Power [43,44].

A total of 38 female footballers from two teams (21 players aged 17 to 29 belonged to an elite team, whereas 17 players aged 13 to 25 belonged to an amateur team) met the following inclusion criteria [16]: (a) having a minimum of 5 years of experience playing football; (b) participating in 3 to 5 games or structured training sessions per week; and (c) belonging to an organised football club and participating in a formal league with regular competitions. Those athletes who did not attend the day of the test (*n* = 2) or those who were not free from injury at the time of testing or had any complaint that would impair their ability to perform the experimental task (*n* = 2) were excluded [16,25].

A total of 34 footballers (age: 19.94 ± 3.94 years; height: 1.63 ± 0.07 m; body mass: 56.26 ± 5.86 kg; BMI: 21.22 ± 2.13 kg/m^2^; federated experience: 6.88 ± 5.73 years; total experience: 10.78 ± 5.97 years) performed the test in the first testing session and were analysed to study the intra- and inter-rater reliability. Then, 15 of those players (age: 21.47 ± 3.31 years; height: 1.61 ± 0.05 m; body mass: 56.02 ± 4.22 kg; BMI: 21.67 ± 1.74 kg/m^2^; federated experience: 9.67 ± 4.62 years; total experience: 13.53 ± 3.56 years) volunteered to participate in a second testing session, which was carried out one week later in the same conditions to establish inter-session reliability.

The study was conducted in accordance with the Ethics of the World Medical Association (Declaration of Helsinki, 7th edition) [45], and the protocol was approved by the Ethics and Research Committee of the University of Murcia (Spain; ID: 2424/2019). All players and legal tutors were informed of the procedure and objectives of the study and signed a written consent form.

### 2.3. Experimental Set-Up and Procedures

**Figure 1:**
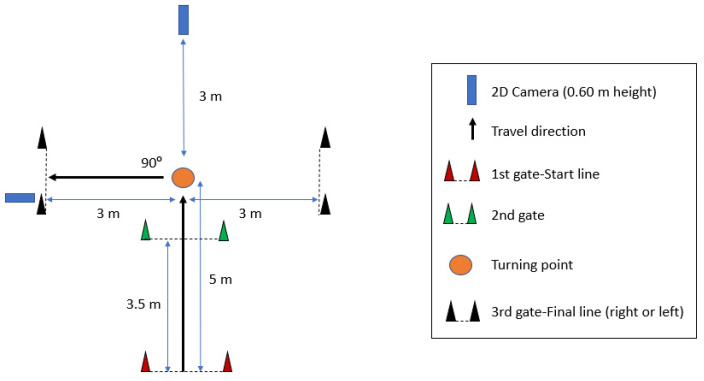
Experimental set-up for the cutting manoeuvre. For cutting to the right, the sagittal camera was positioned on the opposite side, but the procedure was exactly the same.

Similar to the procedures described by Jones et al. (2017) [41] and Dos’Santos, Mc Burnie et al. (2019) [42], the task involved players approaching 5 m towards a turning point. In this case, the turning point was marked with a cone on the floor, subjects had to cut to the left or right at this point and cross a final timing gate positioned 3 m away and 90° from the original direction of travel. Two Panasonic Lumix DMC-FZ200 high-speed cameras (sampling at 100 Hz and 100 FPS; memory card sampling at 90 MB/s) were positioned 3 m away from the turning point in frontal and sagittal planes [39] and on tripods configured approximately at hip-height (0.60 m) [42]. Each cutting trial was simultaneously filmed with both cameras for retrospective 2D qualitative observational analysis (Figure 1). This arrangement allowed data to be collected for both penultimate and final contact [41].

The task was performed on their habitual football field (artificial grass) during a normal training session, and all players were asked to wear their own football boots as well as shorts to facilitate the view of anatomical references [16,17]. A standardised 5 min warm-up based on jogging and self-selected dynamic stretching was carried out prior to participation, as in previous studies [46]. Some practice trials were allowed until the players felt comfortable with the task [16]. After that, six acceptable trials of 90° sidestep cuts per player were recorded [16,42]. Players were asked to perform a minimum of three 90° cuts to the left and another three trials to the right [39]. For each trial, completion time (mean ± SD = 2.33 ± 0.15 s, coefficient of variation (CV) = 6.50%), as well as approach time (mean ± SD = 1.05 ± 0.13 s, CV = 12.49%) were recorded to standardise performance between trials using three sets of timing cells placed at hip height (Microgate Witty photocells, Bolzano, Italy^®^) [42]. The players were initially positioned at 0.5 m behind the start line and were allowed to decide their initial stance leg.

For a trial to be considered valid for posterior analysis, it had to meet the following criteria: (a) cutting to the direction to which they were told to cut to in advance; (b) turning in front of the cone and not over it; and (c) performing the cut at maximum speed, based on observer’s criteria and on the completion time in previous repetitions. The players were instructed to perform the task as fast as they could, and they were encouraged to try harder after each trial to assure a performance similar to a real cutting manoeuvre during a game [25].

### 2.4. Cutting Movement Assessment Score (CMAS)

Table 1 presents the CMAS, which is a qualitative screening analysis tool that has been proved to be a valid tool for the estimation of KAMs during a 90° sidestep cutting task [41,42]. As described by Dos’Santos, McBurnie et al. (2019) [42], the items in this tool are based on the results of previous research investigating the biomechanical factors which determine high KAMs during cutting or which occur in actual ACL injuries observed in videotapes. This tool is further explained by Dos’Santos, McBurnie et al. (2019) [42].

### 2.5. Qualitative Assessment: CMAS

Prior to qualitative screening, all raters attended a pair of online training sessions outlining how to grade the cutting trials using the CMAS (2 h each). The practices were based on the evaluation of pilot video footage to agree on how to observe and score each item of the check list. With support from the supplementary documents created by Dos’Santos, McBurnie et al. (2019) [42], a similar Spanish manual, which contained guidelines to score each item in the CMAS tool, was developed. The manual was also provided to raters with some examples taken from pilot video footage (Supplementary Materials).

The video footage was independently analysed by each researcher using Kinovea (created and developed by Joan Charmant) (0.8.15 version for Windows). This is a free and a user-friendly software which allows raters to play video sequences at slow motion and frame-by-frame. Observers were allowed to play and pause the videos repeatedly to score each check-point of the CMAS tool. It took approximately 4 min, on average, to rate each cutting trial.

Initially, a total of 204 trials (a minimum of 3 trials to each side from 34 players) were screened. To determine inter- and intra-rater reliability, 68 trials (one trial to the right and one to the left from each participant) were randomly selected and analysed. However, the raters agreed to exclude a trial when the technique was not appropriate and the player adopted a round strategy to perform a crossover cut instead of a sidestep cut (*n* = 7) [25], since the CMAS tool was specifically designed to evaluate a sidestep cutting technique.

First, a sport sciences Ph.D. student (rater 1: AAS), as well as an experienced strength and conditioning coach (rater 2: RHG; Ph.D.), independently analysed each trial on two separate occasions one-week apart to examine intra-rater reliability. It must be noted that raters rescored the videos in a random order to prevent recall bias [47]. Second, another experienced strength and conditioning coach (rater 3: AC; Ph.D.) viewed and graded each trial once. Then, the scores of rater 1, rater 2 and rater 3 were compared to establish inter-rater reliability. It must be clarified that all raters had no previous experience with the use of the CMAS tool. Additionally, to estimate the reproducibility of the protocol and the variability of subjects’ performance between sessions, the inter-session reliability was calculated. A total of 15 players from the previous sample volunteered to perform the test again in a second session separated by 7 days from the first. One trial on each side from each subject (*n* = 30) was viewed and scored by rater 1, and these results were compared with the initial scores that rater 1 gave to those players. When a player adopted a crossover strategy in some of the testing sessions, that trial was excluded (*n* = 6).

### 2.6. Statistical Analyses

Intra-class correlation coefficients were determined for the total CMAS score (ICC: model: two-way mixed effects, type: single measure, definition: absolute agreement) to study relative reliability, whereas absolute reliability was verified with the analysis of systematic bias, the typical percentage error of the coefficient of variation (CV_TE_) and the minimal detectable change at a 95% confidence interval (MDC_95_). ICCs were calculated and interpreted based on the following scale: poor (<0.50), moderate (0.50–0.74), good (0.75–0.90) and excellent (>0.90) [48]. A Bland–Altman plot was built to graphically show mean bias and 95% limits of agreement for intra-rater reliability [49].

The typical error of measurement or percentage of change in the mean (systematic bias) was calculated using the spreadsheet from Hopkins (2015) [50]. In addition, differences in means between groups of measures were explored through a paired T-test comparison in SPSS (24.0) to assess the risk of bias.

Measurement precision was determined using the typical percentage error of the coefficient of variation (CV_TE_), while measurement sensitivity was calculated through the minimal detectable change at a 95% confidence interval (MDC_95_), using the spreadsheet from Hopkins (2015) [50]. As previously described by Atkinson and Nevill (1998) [51], CV_TE_ and MDC_95_ were calculated using the log-transformed data to reduce the possible heteroscedasticity of the raw data. The calculations of MDC_95_ and CV_TE_ were conducted through the formula described in the study of Martínez-Romero et al. (2021) [49]. To interpret CV_TE_ values, the arbitrary value (≤10%) suggested by Weir and Vincent (2021) [52] was considered to define good reliability [49].

To study the reliability for each item within the CMAS tool (qualitative variables), percentage agreements (agreements/[agreements + disagreements] × 100) and Cohen’s Kappa coefficients were calculated [42]. Cohen’s Kappa coefficient was considered significant when the *p*-value < 0.05, and its strength was interpreted in the following manner: slight (0.01–0.20), fair (0.21–0.40), moderate (0.41–0.60), substantial or good (0.61–0.80) and almost perfect or excellent (0.81–1.00) [53]. Agreements (%) were interpreted following this scale: excellent (>80%), moderate (51%–79%), and poor (<50%) [42,54]. Fleiss’s Kappa was calculated to study the inter-rater reliability among the three raters and was considered significant when the *p*-value < 0.05. The strength of the Fleiss Kappa was interpreted based on the following scale: poor (<0.40), acceptable (0.40–0.60), good (0.61–0.75) and excellent (>0.75) [55].

All statistical analyses were performed using the Statistical Package for Social Science (IBM Corp.; IBM SPSS Statistics for Windows, version 24.0, Armonk, NY, USA) and an online spreadsheet (www.sportsci.org) (accessed on 15 June 2020). An alpha level of 0.05 was defined to consider statistically significant results.

## 3. Results

All statistical analyses were independently performed with the sample of elite players and the sample of amateur players; however, only the results for the total sample are presented, because no differences in reliability based on competitive level were found.

### 3.1. Intra- and Inter-Rater Reliability

Table 2 shows the intra-rater and inter-rater reliability data for the total CMAS score when analysing the 61 cases from the sample of 34 female footballers. Poor correlations with ICCs ranging from 0.11 to 0.45 were found when inter-rater paired comparisons were explored. In contrast, both rater 1 and rater 2 demonstrated moderate (ICC = 0.71; ICC = 0.61) intra-rater reliability for the CMAS score evaluation. However, even for intra-rater reliability, the CV_TE_ values were greater than 10% for both rater 1 (CV_TE_ = 17.4%) and rater 2 (CV_TE_ = 18.7%). In addition, there was a significant difference in the means between the scores given by rater 1 (*p* < 0.05), with a systematic bias of 5.6%. Furthermore, when the CMAS score was analysed by the same rater on different occasions, it was found that the MDC_95_ was higher than 30% in both cases (rater 1: MDC_95_ = 34.5%; rater 2: MDC_95_ = 37.0%).

**Figure 2:**
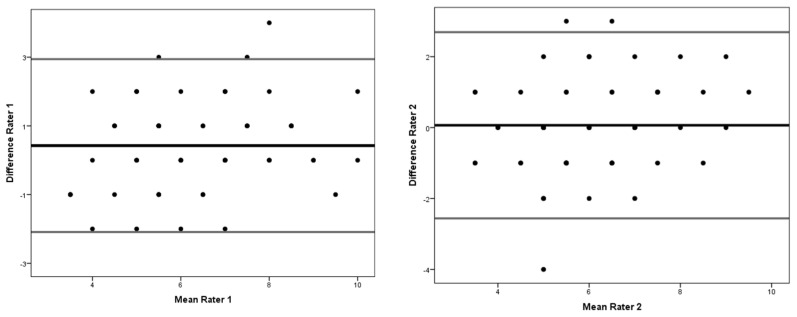
Bland–Altman plots to graphically show intra-rater reliability for rater 1 and rater 2, respectively. The *y*-axis shows the mean of the differences between the CMAS scores given in the first and second evaluations by rater 1 and rater 2, respectively, as well as the 95% confidence intervals for these means. The *x*-axis shows the mean of the two scores given for each of the cases analysed.

Figure 2 represents the concordance and agreement of the measures of the CMAS scores given in the first and second evaluation by each rater for the 61 cases.

Intra-rater as well as inter-rater reliability data (percentage of agreement and Cohen’s Kappa coefficient) for each criterion of the CMAS tool are presented in Table 3. Rater 1 and rater 2 presented significant and moderate-to-good intra-rater reliability for most items of the CMAS tool (Table 3). Both raters displayed good intra-rater reliability for items 2, 4 and 6 (k = 0.64–0.77) with excellent agreement (86.9% to 93.5%). Rater 1 showed an excellent kappa coefficient (0.85) and agreement (93.5%) for item 9 between the first and second score, while showing fair intra-rater reliability for items 1, 3 and 5 (k = 0.28–0.39; 67.2% and 85.3%). Additionally, rater 2 displayed only a fair reliability for item 7 (k = 0.40; agreement = 75.4%), but moderate-to-good reliability (k = 0.43–0.77) was demonstrated for the other items.

For inter-rater reliability, all paired comparisons showed a slight-to-fair, and not always significant, reliability for most items of the CMAS tool. However, raters 1 and 2 presented moderate inter-rater reliability for items 4, 6 and 7 (k = 0.41–0.45 and a moderate to excellent agreement 73.9%–85.2%). Likewise, raters 1 and 3 only showed moderate inter-rater reliability in scoring item 6 (k = 0.55; agreement = 75.5%). In fact, results from the Fleiss’ Kappa analysis only demonstrated a significant and acceptable reliability among the three raters for item 6 (Fleiss-k = 0.41), whereas poor inter-rater reliability was obtained for the rest of the items in the tool (Fleiss-k ranging from −0.17 to 0.28).

### 3.2. Inter-Session Reliability

When rater 1 analysed and scored the 24 cases from the 15 players who performed the test on two different occasions, poor correlation was found between the CMAS scores given to the players in each session (mean score 1 = 6.0 ± 1.7; mean score 2 = 5.4 ± 1.3; systematic bias (%) = −9.4 (CI 95%: −18.5–0.9); *p*-value (paired T-test) = 0.092; CV_TE_ (%) = 24.1 (CI 95%: 19.1–33.1); ICC = 0.39 (CI 95%: 0.06–0.64); MDC_95_ (%) = 47.7 (CI 95%: 37.8–65.6)).

In relation to the test–retest reliability of the CMAS tool criteria, only items 1, 2 and 6 showed significant inter-session reliability (Table 4). Specifically, items 1 and 6 presented moderate reliability (item 1: k = 0.50 and agreement = 87.5%; item 6: k = 0.59 and agreement = 83.4%), while item 2 showed an excellent level of correlation (k = 0.90) and agreement (95.9%).

## 4. Discussion

The examination of the cutting-movement quality of female footballers in a sport-specific context and using accessible tools is particularly relevant and could constitute an essential pillar among screening protocols to evaluate potential injury risk in women’s football. Evaluating movement quality can help inform injury mitigation training and also help in guiding the recovery and rehabilitation processes of female players rehabilitating from an ACL injury [38]. Thus, the present study was carried out to check if the 90° COD protocol and the qualitative screening tool called the Cutting Movement Assessment Score (CMAS tool)—designed and proposed by Dos’Santos, McBurnie et al. (2019) [42]—could also be reliable when applied in a more real context with a specific sample of female footballers.

### 4.1. Methodological Adaptations for Applied Settings

As differences in methods and testing procedures might influence the reliability results, some modifications in methodology applied in the present study with respect to the original investigation [42] need to be clarified and discussed. To make the test application and evaluation totally accessible for strength and conditioning coaches and female football Spanish teams, some methodological adaptations were performed. First, athletes who had previously suffered an ACL injury, and had completely recovered from it, were not excluded in this study, as these players would also need to be evaluated in a real situation. Second, the test was carried out on a football field during a regular training session, and the players were wearing their usual boots and football kit. In contrast, Dos’Santos, McBurnie, Comfort and Jones (2019) [56] applied the COD task on an indoor hardwood court with 19 male youth footballers.

The third difference was that the turning point was marked with a cone and players were told to turn at this point, but were not forced to perform the final foot contact (FFC) with a particular leg and within a specific area. However, that could allow the players to adopt a round strategy to perform a crossover cut instead of a sidestep cut at the time of testing. For that reason, when the recorded technique was not appropriate, the trial had to be excluded later from the analysis (*n* = 7), because the CMAS tool was specifically designed to evaluate the sidestep cutting technique [25]. In real conditions, this would mean that not all players would have complete COD evaluation. Conversely, in the study of Dos’Santos, McBurnie, Comfort and Jones (2019) [56], a mark on the floor was used to indicate the turning point and trials were directly discarded at the time of testing when players cut prematurely or when they performed a crossover cut.

In addition, although too many instructions might alter the real cutting strategy, it is important to standardise performance somehow to obtain consistent and reliable results [39,57,58,59,60]. Therefore, drawing a small specific area on the field where the FFC should be performed with a particular leg but telling athletes to look straight ahead to avoid targeting this point, might be suggested as a solution [57]. In that way, coaches would ensure that every player would perform the COD using a sidestep cutting strategy at the time of testing. Indeed, in a recent meta-analysis, it was shown how methodological changes can influence and improve the reliability of a field-based test [61]. In fact, Everard et al. (2019) [47] found excellent intra- and inter-rater reliability using the Landing Error Scoring System (LEES) to evaluate a landing task which involved athletes landing in a specific point drawn on the floor.

On the other hand, noting that women’s football teams in Spain have few available means and resources, it was decided to keep the initial two cameras (frontal and sagittal) used in the preliminary study of Jones et al. (2017) [41], as an orthogonal perspective (90°) has been recommended for motion analysis in Kinovea [62]. Therefore, the third additional 45° camera used in the study of Dos’Santos, McBurnie et al. (2019) [42] and in the study of Dos’Santos, McBurnie, Comfort and Jones (2019) [56] was removed. It must be pointed out that only two 2D video cameras (frontal and sagittal) were used in a recent study with 34 recreational and elite footballers for the kinematic analysis of a similar 90° sidestep cutting task [40].

The last methodological variation was related to the analysis. As players performed the COD to both sides, a trial to each side from each athlete was selected and evaluated to study intra-rater and inter-rater reliability. It must therefore be noted that cutting trials to each side would need to be assessed separately to explore inter-limb differences [63,64,65].

### 4.2. Intra- and Inter-Rater Reliability

The main purpose of this study was to determine the intra- and inter-rater reliability using the CMAS tool to assess 90° sidestep cutting technique. The cutting trials of two Spanish female football teams were therefore analysed by three different raters. With respect to the reliability of the CMAS score, poor correlations (with ICCs ranging from 0.11 to 0.45) were found among each pair of raters. Although Dos’Santos, McBurnie et al. (2019) [42] found better results for the inter-rater reliability of the CMAS score (ICC = 0.69) with only a one-hour training session, these authors also found only fair or poor inter-rater reliability for some items of the tool. Other qualitative screening tools, such as the tuck jump assessment, have also shown disparity in reliability results across studies when the reliability was analysed by research groups other than the original group that created the tool [66,67]. These differences might be due to better prior familiarisation of the original group with the testing procedures.

For that reason, it seems that, in general, more than two training sessions for raters might be needed to explain, discuss and agree on the different observation criteria for each item of the check-list. In a recent study, which investigated the reliability of an observation tool (the Basic Functional Assessment) to evaluate fundamental movement patterns, it was outlined that more training sessions for observers might lead to better inter-rater reliability results [59]. Some reliability studies using qualitative observational screening tools have also obtained poor reliability results with only one or two training sessions for observers [68,69].

In addition, although the observers demonstrated moderate (ICC = 0.71 and ICC = 0.61) intra-rater reliability for the CMAS score evaluation, even for intra-observer reliability, the precision of measurement was greater than the accepted value of 10% for both raters (CV_TE_ ranged from 17.4% to 18.7%) [52]. In fact, poor sensitivity was found when the CMAS score was analysed by the same rater on different occasions, with a minimum detectable change (MDC_95_) higher than 30% in both cases. This means that a change of at least 3 to 4 points out of 11 would be needed to determine a real improvement in COD technique using the CMAS score. However, a recent experimental study exploring the effects of a six-week change of direction training on cutting movement quality using the CMAS tool with 19 male professional youth footballers showed a mean improvement of only 1.5 points and 2.2 points out of 11 for COD to the right and to the left, respectively [56].

Apart from that, a high risk of bias was also found, as rater 1 (Ph.D. student) had a systematic bias of 5.6%, with a significant difference in means between the first and second score given (*p* < 0.05). For that reason, a standardised previous training period is recommended in which each observer could independently rate a greater quantity of pilot trials to familiarise the raters with the use of the CMAS tool [70]. Indeed, some studies have pointed out the importance of familiarisation sessions to reduce the learning effect and to improve measurement reliability [49,61,71]. Some studies have also noted differences in reliability results depending on the rater’s level of experience [72].

In the intervention study by Dos’Santos, McBurnie, Comfort and Jones (2019) [56], intra- and inter-rater reliability for CMAS score and criteria were also studied, and the authors determined excellent intra- and inter-rater reliability for both the CMAS total score and for the variables of the tool. The different results found in the study of Dos’Santos, McBurnie, Comfort and Jones (2019) [56] with respect to the current investigation might be due to methodological variations between studies. As Dos’Santos, McBurnie, Comfort and Jones (2019) [56] carried out an experimental study, it was not specifically designed to determine the reliability of the CMAS tool. Even so, it must be mentioned that the smallest detectable differences for the CMAS scores found in the study of Dos’Santos, McBurnie, Comfort and Jones (2019) [56] were also very high (21.7 to 42.6%).

In contrast, when intra-rater reliability was independently explored for each item in the current study, it was found that raters presented a significant, as well as moderate-to-good intra-rater reliability for most items of the CMAS tool. Similarly, Dos’Santos, McBurnie et al. (2019) [42] found excellent intra-rater reliability for all variables of the CMAS tool. It might therefore be interpreted that the CMAS tool could be used to evaluate the effect of interventions on COD technique, as long as the first and second assessments were carried out by the same rater, and if only the specific improvement in each deficit was independently considered.

Nevertheless, some items of the CMAS did not reach moderate intra-rater reliability in the present study. For instance, rater 1 had only fair intra-rater reliability for items 1 “Clear PFC Braking”, 3 “Hip… initial internally rotated…” and 5 “No neutral foot position”, while rater 2 obtained fair reliability scoring item 7 “Trunk upright or leaning back”. This aspect could be improved with more precise or clearer instructions about the visual references that must be taken into account to score each manifestation [68].

For instance, in item 3, raters had to decide if the hip was in an internally rotated position at initial contact; however, rotational movements are difficult to observe in 2D video recordings [22,40,73]. In the study by Dos’Santos, McBurnie et al. (2019) [42], reflective markers were placed on players’ anatomical reference points for 3D analysis, while no markers were used on athletes’ skin in the present research. This visual reference placed on the players’ thigh might explain why those authors obtained perfect intra-rater reliability for item 3 (k = 1.00). Despite this, it must be mentioned that Dos’Santos, McBurnie et al. (2019) [42] found slight and poor reliability among raters while scoring hip internal rotation (kappa ranging from 0.067 to 0.194).

Likewise, the evaluation of an inwardly or externally rotated foot position (item 5) could have been made more precise by drawing a line on the floor in the original direction of travel to indicate a neutral foot position, as external references might lead to better reliability results [61,74]. Similarly, to evaluate trunk flexion displacement (item 7), an additional visual reference might be given. For instance, adequate trunk flexion could be considered when some point of the head goes beyond an imaginary vertical line drawn from the supporting leg’s knee-joint centre, or simply when trunk flexion increases from initial contact through the weight acceptance phase [47]. It needs to be acknowledged that the authors have recently provided further information to assist in the qualitative screening of the items with clearer operation guidelines [75].

As for the inter-rater reliability for each item, all paired comparisons showed not only a slight-to-fair (k ≤ 0.40) relationship between measures, but also a non-significant (*p* > 0.05) reliability between observers in rating most check-points of the CMAS tool. It must be noted that the content validity of the CMAS tool has not previously been studied, which might have affected the results obtained.

In item 1 “Clear PFC Braking”, observers were instructed to rate the deceleration strategy of the players. In the present study, raters had to pay attention to the backward inclination of the trunk, anterior foot placement and heel contact as indicators of a good braking strategy. However, it is not clarified within the tool if the athlete should show the three aspects to have their braking strategy rated as “good” or if they just had to show at least one or two of those indicators.

Likewise, observers are asked to evaluate knee-joint motion in item 8, with the instruction of rating a knee flexion of less than 30° as limited. On the one hand, the angle to take into account should be specified, as knee flexion has been explored through different angles [3,34]. On the other hand, as knee flexion is not observed in a pure sagittal view due to the camera position, it might be better to ask raters if they observe a good sagittal knee absorption of forces on the whole [47,76], rather than giving an angular reference, which could be misinterpreted because of perspective [23,62]. For that reason, exploring the content validity of this tool through the judgement of a minimum of ten experts in the field is therefore recommended [59,77,78].

Content validity should be studied not only in terms of the adequacy and adaptation of the items, definitions and instructions included within the manual of the tool, but also in terms of their relevance to the instrument, as described in previous studies [59,77,78].

Items 4 “Initial knee valgus position”, 6 “Frontal plane trunk position” and 7 “Trunk upright or leaning back” obtained the best reliability results when paired comparisons between raters were analysed, whereas significant and acceptable reliability among the three raters was only found for item 6. According to these results, it was similarly found that both raters displayed good intra-rater reliability in scoring items 2 “Wide lateral leg plant”, 4 and 6, as well as moderate-to-excellent reliability rating item 9 “Excessive knee valgus motion” and fair-to-good reliability scoring item 7.

Raters agreed to evaluate initial knee valgus position (item 4) with reference to the knee abduction angle at initial contact, as has commonly been used to calculate KAM [12,18,24,79]. This could have led to better reliability results for item 4. In terms of rating the frontal plane trunk position (item 6), it must be noted that this is the only item with more than two categories. This fact could have made its evaluation clearer, as it might have required a more precise definition or criterion to differentiate each category [80,81,82].

A quantitative reference in metres was given as guidance to rate lateral leg plant distance (item 2); however, Weir et al. (2019) [39] found that only angular measures were reliable enough to analyse COD mechanics. Thus, an angular reference for rating this variable might be more appropriate and could improve inter-rater reliability results for item 2. In the same way, inter-rater reliability for the evaluation of knee valgus motion (item 9) might be improved with a more objective angular reference, such as the knee abduction angle, which has been shown to have a greater impact on the KAM than the hip adduction angle [18,40].

### 4.3. Inter-Session Reliability

A second aim was to ascertain the inter-session reliability to estimate the variability of players’ COD technique among different sessions. Poor reliability was found between the CMAS scores given to the players in each session. Additionally, it was observed that only items 1 “Clear PFC Braking”, 2 “Wide lateral leg plant” and 6 “Frontal plane trunk position” showed significant inter-session reliability (moderate-to-excellent association). This might mean that only those manifestations are consistently shown and detected across time; however, these results must be interpreted with caution, as only 15 players were included in the performance for inter-session analysis. Furthermore, intra-rater bias could also have affected the variability observed with respect to players’ performance at different sessions. Read et al. (2016) [36] also found that only the knee valgus item showed substantial agreement and strong reliability between trials when analysing the inter-session reliability of the tuck-jump injury-risk screening assessment in elite male youth footballers.

McLean et al. (1999) [83] reported that male and female athletes with lower levels of experience showed greater kinematic variability in global change-of-direction mechanics. This greater motor variability in a sport context denotes a high motor ability to adapt to different situations. However, extreme variability in different repetitions of the same test or global task has commonly been associated with low levels of experience or physical preparation. In this sense, McLean et al. (1999) [83] stated that experience level was the only factor that had a significant effect on the kinematic variability of the knee joint. According to this, it could be hypothesised that better inter-session reliability results would have been obtained if the test had been applied to professional athletes instead of amateur or elite athletes.

Pollard et al. (2015) [84] found that female footballers who had undergone ACL reconstruction and returned to play exhibited increased lower extremity movement variability during a sport-specific task such as sidestep cutting. As players who had suffered and recovered from an ACL injury were not excluded in the present study, this also may have affected inter-session reliability results.

Another point is that only a repetition to the right and to the left were selected from each player for posterior analysis in the present study. This is similar to the study by Dos’Santos, McBurnie et al. (2019) [42], as they only analysed one trial from each athlete to study the reliability of the CMAS tool. The inter-session reliability data shown in the current study suggest that taking the average of at least two trials might improve the bias based on subject variability; however, this would imply a greater investment of time for analysis by coaches, which could hinder the applicability of the tool.

As for limitations, it must be pointed out that taking trials from each side as different cases for the analysis could have affected heterogeneity and altered reliability results [85]. Nevertheless, it was decided to take into account COD on both sides to simulate real conditions, as coaches would need to apply the test in both directions to evaluate inter-limb asymmetries [63,64,65].

Future research should explore the content validity of the CMAS tool as well as give quantitative references for each criterion to create more objective observable categories. As suggested by previous studies, to complete the process of validation of an observational instrument focused on technical actions in sports, a qualitative and quantitative assessment of the content validity should be performed by at least 10 experts in the field [59,77,78,82]. This might help to make the items, definitions and instructions included within the manual of the CMAS tool more precise and clearer. In addition, although the sample size was appropriate to obtain relevant statistical results, future studies should analyse the reliability of the CMAS tool with a larger sample and include professional players as well.

For future application of the test in a field-based context, usage of a small area drawn on the floor instead of a cone as the turning point’s reference could facilitate the performance of the COD task with a sidestep cutting strategy. Secondly, a marker on players’ thighs and a line drawn along the original direction of travel might serve as visual references for observational analysis. Finally, some standardised extra training sessions to familiarise observers with independently rating a considerable quantity of pilot trials is highly recommended.

## 5. Conclusions

Regarding CMAS score, poor reliability among each pair of raters was found, as well as moderate reliability when the score was given by the same rater at different moments, but with too high a minimum detectable change, because at least 3 to 4 points out of 11 in the CMAS score would be needed to consider a real change in COD technique. On the other hand, raters presented significant, as well as moderate-to-good intra-rater reliability for most items of the CMAS tool. In fact, both raters achieved good intra-rater reliability in scoring items 2 “Wide lateral leg plant”, 4 “Initial knee valgus position” and 6 “Frontal plane trunk position”, as well as moderate-to-excellent reliability in rating item 9 “Excessive knee valgus motion”. However, there was only a slight-to-fair relationship and a non-significant reliability between observers in rating most check-points of the tool. In addition, poor reproducibility of the protocol was found, with poor correlations in the scores given by the same observer between sessions. For these reasons, more objective (cut-off points) and clearer definitions for each criterion within the CMAS, as well as a longer and standardised training period for novel observers, would be highly recommended to improve reliability using this tool in an applied context with women footballers.

## Figures and Tables

**Table 1:** Cutting Movement Assessment Score tool.

Camera	Variable	Observation	Score
Penultimate foot contact			
Side	Clear PFC braking strategy (at initial contact)	Y/N	Y = 0/N = 1
	Backward inclination of the trunk Large COM to COP position—anterior placement of the foot Effective deceleration—heel contact PFC		
Final foot contact			
Front	Wide lateral leg plant (at initial contact)	Y/N	Y = 2/N = 0
	Approximately > 0.35 m—dependent on subject’s anthropometrics		
Front	Hip in an initial internally rotated position (at initial contact)	Y/N	Y = 1/N = 0
Front	Initial knee valgus position (at initial contact)	Y/N	Y = 1/N = 0
Front/Side	Foot not in neutral foot position (at initial contact)	Y/N	Y = 1/N = 0
	Inwardly rotated foot position or externally rotated foot position (relative to original direction of travel)		
Front	Frontal plane trunk position relative to intended direction (at initial contact and over WA phase)	L/TR/U/M	L/TR = 2/U = 1/M = 0
	Lateral (L) or trunk rotated (TR) towards stance limb Upright (U) Medial (M)		
Side	Trunk upright or leaning back throughout contact (at initial contact and over WA phase)	Y/N	Y = 1/N = 0
	Inadequate trunk flexion displacement		
Side	Limited knee Flexion during final contact (over WA)	Y/N	Y = 1/N = 0
	Knee flexion ≤ 30° (stiff)		
Front	Excessive Knee “valgus” motion during contact (over WA)	Y/N	Y = 1/N = 0
		Total Score	/11

PFC: Penultimate foot contact; COM: Centre of mass; COP: Centre of pressure; WA: Weight acceptance phase; TR: Trunk rotation; Y: Yes; N: No; L: Lateral; U: Upright; M: Medial.

**Table 2:** Intra-rater and inter-rater reliability of the CMAS score (*n* = 61 cases).

Statistic	Intra-Rater 1	Intra-Rater 2	Inter-Rater 1 vs. 2	Inter-Rater 1 vs. 3	Inter-Rater 2 vs. 3
Score 1 (Mean ± SD)	6.6 ± 1.9	6.1 ± 1.7	6.6 ± 1.9	6.6 ± 1.9	6.1 ± 1.7
Score 2 (Mean ± SD)	6.1 ± 1.6	6.0 ± 1.4	6.1 ± 1.7	4.9 ± 1.7	4.9 ± 1.7
Systematic bias (%) [95% CI]	−5.6 * [−10.1 to −0.9]	0.0 [−5.0 to 5.3]	−7.0 [−14.8 to 1.4]	−26.5 † [−31.9 to −20.6]	−20.9 † [−27.6 to −13.6]
CV_TE_ (%) [95% CI]	17.4 [15.0 to 20.8]	18.7 [16.1 to 22.4]	33.2 [28.4 to 40.2]	29.1 [24.9 to 35.1]	33.8 [28.8 to 40.9]
MDC_95_ (%) [95% CI]	34.5 [29.7 to 41.2]	37.0 [31.9 to 44.3]	65.7 [56.1 to 79.5]	57.6 [49.3 to 69.6]	66.8 [57.1 to 80.9]
ICC [95% CI] (strength)	0.71 [0.58 to 0.80] (moderate)	0.61 [0.46 to 0.73] (moderate)	0.11 [−0.11 to 0.31] (poor)	0.45 [0.26 to 0.60] (poor)	0.24 [0.03 to 0.43] (poor)

CMAS: Cutting Movement Assessment Score; Significance (paired T-test): * *p* < 0.05; † *p* < 0.001; ICC strength: poor (<0.50), moderate (0.50–0.74), good (0.75–0.90), and excellent (>0.90) [48].

**Table 3:** Intra-rater and inter-rater reliability of CMAS tool criteria (*n* = 61 cases).

CMAS Tool Criteria	Intra-Rater 1		Intra-Rater 2		Inter-Rater 1 vs. 2		Inter-Rater 1 vs. 3		Inter-Rater 2 vs. 3		Inter-Rater 1 vs. 2 vs. 3
	% Agreement (Strength)	k (Level)	% Agreement (Strength)	k (Level)	% Agreement (Strength)	k (Level)	% Agreement (Strength)	k (Level)	% Agreement (Strength)	k (Level)	Fleiss-k (Level)
1. Clear PFC Braking	85.3 (excellent)	0.388 * (fair)	86.8 (excellent)	0.525 † (Mod)	82.0 (excellent)	0.164 (slight)	83.6 (excellent)	0.193 (slight)	85.2 (excellent)	0.316 * (fair)	0.225 * (poor)
2. Wide lateral leg plant	88.5 (excellent)	0.761 † (good)	88.4 (excellent)	0.672 † (good)	52.5 (moderate)	0.167 (slight)	50.8 (moderate)	0.194 * (slight)	75.4 (moderate)	0.334 * (fair)	0.144 (poor)
3. Hip in an initial internally rotated position	67.2 (moderate)	0.303 * (fair)	91.8 (excellent)	0.688 † (good)	60.7 (moderate)	0.082 (slight)	65.6 (moderate)	0.323 * (fair)	55.7 (moderate)	0.161 (slight)	0.152 * (poor)
4. Initial knee valgus position	90.2 (excellent)	0.642 † (good)	93.5 (excellent)	0.740 † (good)	85.2 (excellent)	0.439 † (Mod)	68.8 (moderate)	0.225 * (fair)	70.5 (moderate)	0.274 * (fair)	0.277 † (poor)
5. No neutral foot position	83.6 (excellent)	0.281 * (fair)	73.8 (moderate)	0.432 † (Mod)	72.2 (moderate)	0.319 * (fair)	54.1 (moderate)	0.154 (slight)	59.0 (moderate)	0.203 (slight)	0.165 * (poor)
6. Frontal plane trunk position relative to intended direction	86.9 (excellent)	0.745 † (good)	93.5 (excellent)	0.773 † (good)	73.9 (moderate)	0.445 † (Mod)	75.5 (moderate)	0.547 † (Mod)	64.0 (moderate)	0.275 † (fair)	0.410 † (acceptable)
7. Trunk upright or leaning back throughout contact	88.6 (excellent)	0.649 † (good)	75.4 (moderate)	0.399 * (fair)	77.1 (moderate)	0.410 * (Mod)	83.6 (excellent)	0.381 † (fair)	70.5 (moderate)	0.083 (slight)	0.274 † (poor)
8. Limited knee flexion during final contact	80.4 (excellent)	0.598 † (Mod)	91.8 (excellent)	0.620 † (good)	54.1 (moderate)	0.114 (slight)	55.8 (moderate)	0.072 (slight)	16.4 (poor)	0.010 (slight)	−0.166 * (poor)
9. Excessive knee valgus motion during contact	93.5 (excellent)	0.848 † (excellent)	78.7 (moderate)	0.560 † (Mod)	54.1 (moderate)	0.114 (slight)	59.0 (moderate)	0.280 * (fair)	62.3 (moderate)	0.226 (fair)	0.169 * (poor)
Average	84.86	0.58	85.97	0.60	67.98	0.25	66.31	0.26	62.11	0.21	0.16

CMAS: Cutting Movement Assessment Score; PFC: Penultimate Foot Contact. *p*-value: * *p* < 0.05; † *p* < 0.001; Strength of agreement: excellent (≥80%); moderate (50–79%); poor (<50%) [42,54]; Level of Cohen’s k correlation: slight (0.01–0.20); fair (0.21–0.40); Mod= moderate (0.41–0.60); good (0.61–0.80); excellent (0.81–1.00) [53]; Level of Fleiss-k correlation: poor (<0.40); acceptable (0.40–0.60); good (0.61–0.75); excellent (>0.75) [55].

**Table 4:** Inter-session reliability (session 1 vs. session 2 analysed by rater 1) of CMAS tool criteria (*n* = 24 cases).

CMAS Tool Criteria	Session 1 vs. Session 2	
	Percentage of Agreement (Strength)	Cohen’s Kappa (Level of Correlation)
1. Clear PFC Braking	87.5 (excellent)	0.500 * (Mod)
2. Wide lateral leg plant	95.9 (excellent)	0.903 † (excellent)
3. Hip in an initial internally rotated position	54.2 (moderate)	0.096 (slight)
4. Initial knee valgus position	58.4 (moderate)	−0.071 (none)
5. No neutral foot position	50.0 (moderate)	−0.083 (none)
6. Frontal plane trunk position relative to intended direction	83.4 (excellent)	0.590 * (Mod)
7. Trunk upright or leaning back throughout contact	75.0 (moderate)	0.395 (fair)
8. Limited knee flexion during final contact	54.2 (moderate)	−0.158 (none)
9. Excessive knee valgus motion during contact	75.0 (moderate)	0.339 (fair)

CMAS: Cutting Movement Assessment Score; PFC: Penultimate Foot Contact; *p*-value: * *p* < 0.05; † *p* < 0.001; Strength of agreement: excellent (≥80%); moderate (50–79%); poor (<50%) [54]; Level of correlation: slight (0.01–0.20); fair (0.21–0.40); Mod= moderate (0.41–0.60); good (0.61–0.80); excellent (0.81–1.00) [53].

## Data Availability

The data presented in this study are available within the article.

## Supplementary Materials

The following supporting information can be downloaded at: https://www.mdpi.com/article/10.3390/ijerph19074143/s1.
