# Supplementary material for: Reliability of a Qualitative Instrument to Assess High-Risk Mechanisms during a 90° Change of Direction in Female Football Players
Source: Int J Environ Res Public Health. 2022 Mar 31;19(7):4143. doi: 10.3390/ijerph19074143 (PMC8999027; doi:10.3390/ijerph19074143)

MANUAL ANÁLISIS “CUTTING MOVEMENT ASSESSMENT SCORE (CMAS)” EN COD 90º (side-step)

Penúltimo contacto previo al COD (PFC)

Sagital	**Clara estrategia de deceleración en el penúltimo contacto** (en el momento del contacto inicial)	**Nota explicativa**
	• Inclinación del tronco hacia atrás<br>• Gran distancia desde el COM al COP- posición anterior del pie<br>• Deceleración efectiva – contacta el talón en el penúltimo contacto	Para marcar “Sí” debe cumplir los tres puntos especificados

Sí=0

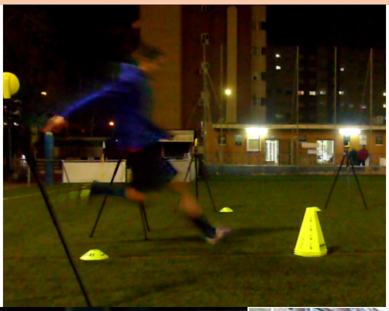

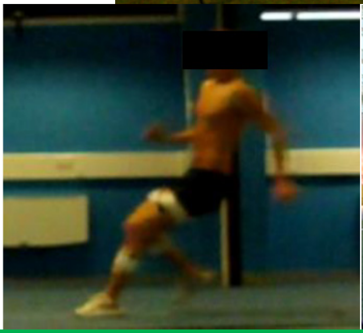

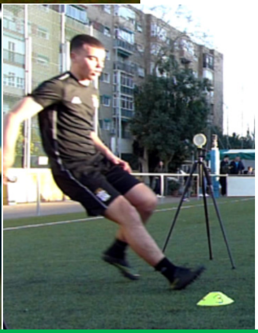

No=1

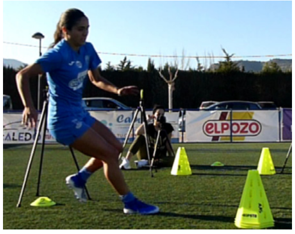

*No muestra inclinación del tronco hacia atrás

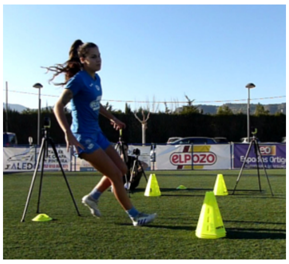

*La distancia COM-COP es pequeña y no hay inclinación del tronco hacia atrás

Contacto final previo al COD (FFC)

Frontal	**Gran amplitud en el apoyo lateral** (en el momento del contacto inicial)	**Nota explicativa**
	• Aprox. > 0.35 m – dependiendo de la antropometría del sujeto (si es niño o es muy bajito cambiar a >0.25 m)	Se considera una gran amplitud en el apoyo lateral cuando la distancia entre la cadera (de la pierna de apoyo) y el pie de apoyo es mayor a 0,35 m en el momento del contacto inicial.

Sí=2

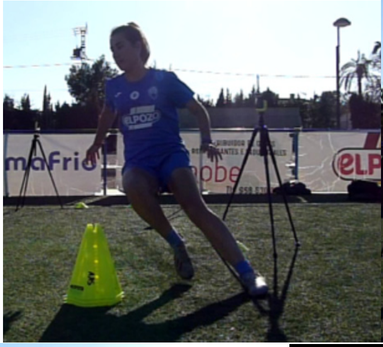

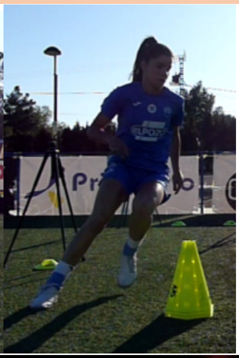

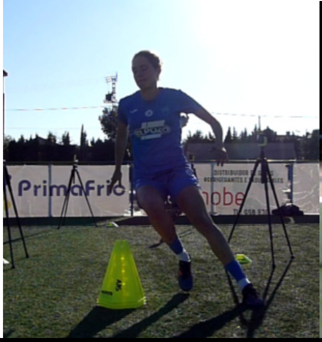

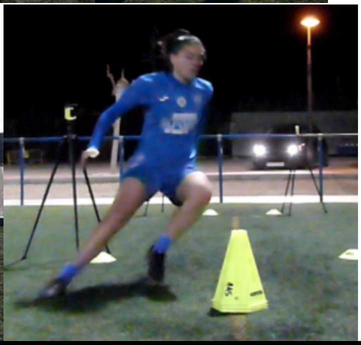

No=0

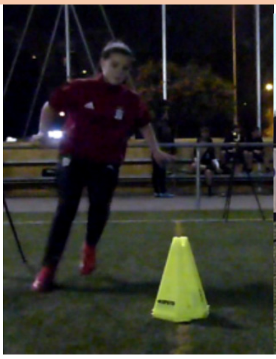

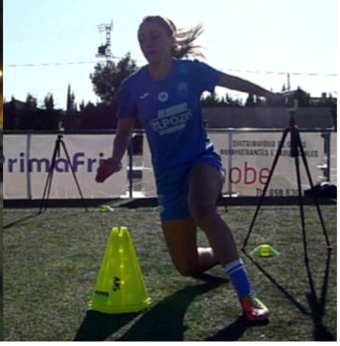

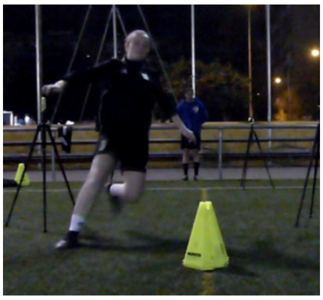

Frontal	**Cadera en posición de rotación interna inicial** (en el momento del contacto inicial)	**Nota explicativa**
	• Se observa una rotación interna inicial del muslo	Se recomienda retroceder a unos frames previos al contacto inicial y observar si se produce rotación interna del muslo

MANUAL ANÁLISIS "CUTTING MOVEMENT ASSESSMENT SCORE (CMAS)" EN COD 90º (side-step)

Sí=1	No=0
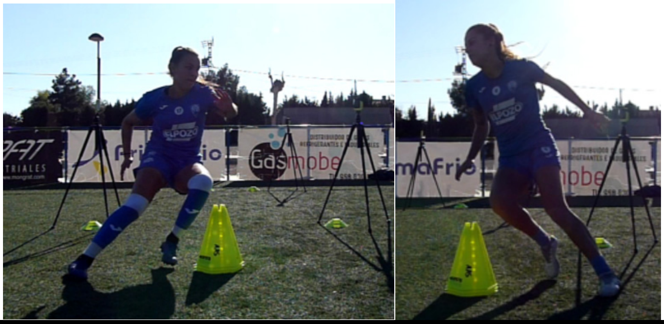	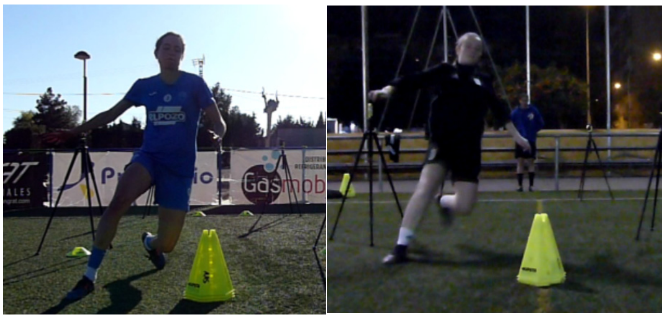

**Frontal**	**Valgo de rodilla inicial** (en el momento del contacto inicial)	**Nota explicativa**
	• Posicionamiento medial de la rodilla en el contacto inicial	Si hay duda, puede tomarse como referencia el ángulo FPPA (FPPA< 170º)

Sí=1	No=0
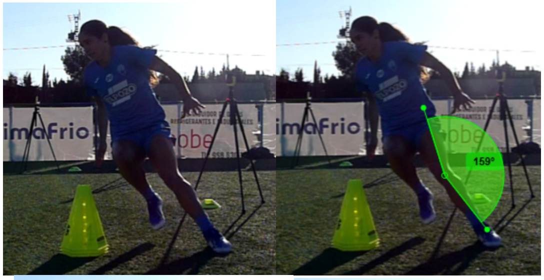	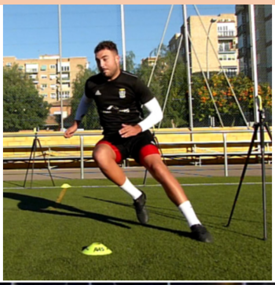
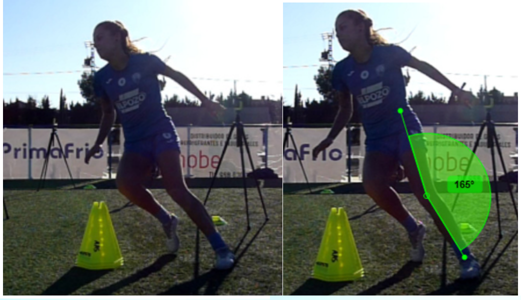	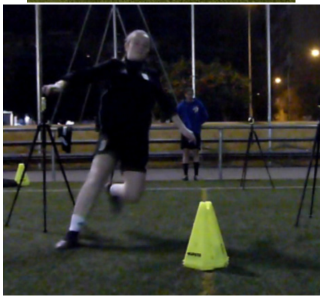
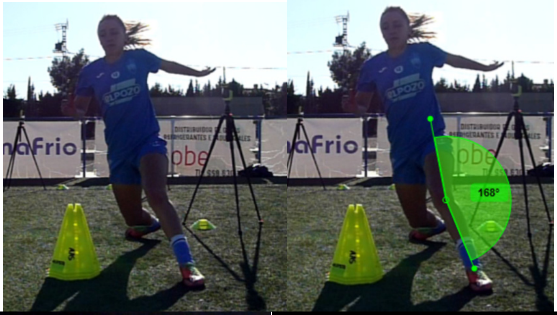	
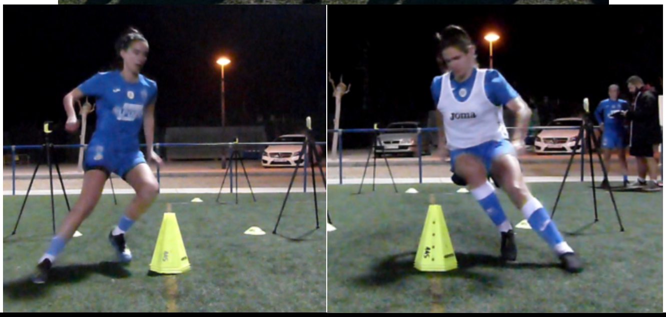	

**Frontal/Sag.**	**El pie no está orientado en posición neutra** (en el momento del contacto inicial)	**Nota explicativa**
	• Se produce rotación interna o externa del pie (en relación a la dirección del recorrido inicial de la primera fase de carrera)	Puede trazarse una línea de referencia para marcar la dirección del recorrido inicial de la carrera

Sí=1	No=0

MANUAL ANÁLISIS "CUTTING MOVEMENT ASSESSMENT SCORE (CMAS)" EN COD 90º (side-step)

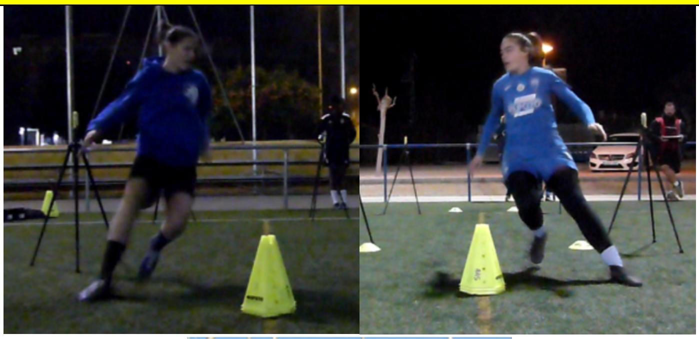

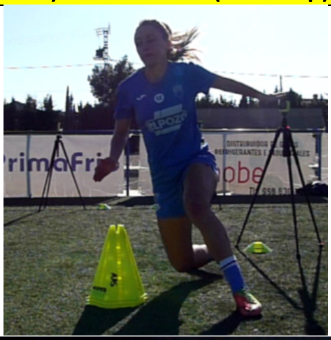

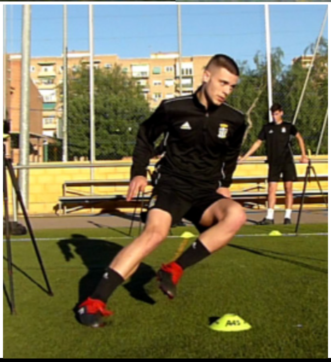

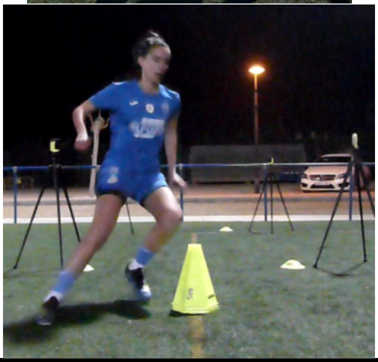

Frontal	**Posición frontal del tronco con relación a la nueva dirección de carrera** (en el momento del contacto inicial y a lo largo de la fase WA)	**Nota explicativa**

- Lateral (L) o tronco rotado (TR) hacia la pierna de apoyo
- Upright (U) o erguido, en vertical
- Medial (M) o hacia la nueva dirección de carrera

Puede usarse la posición de los hombros como guía (trazar una línea de referencia en kinovea)

L=2

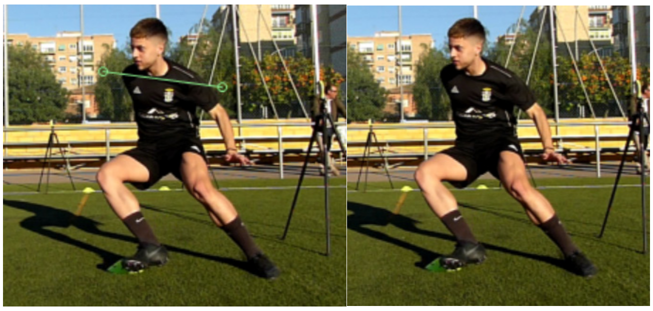

U=1

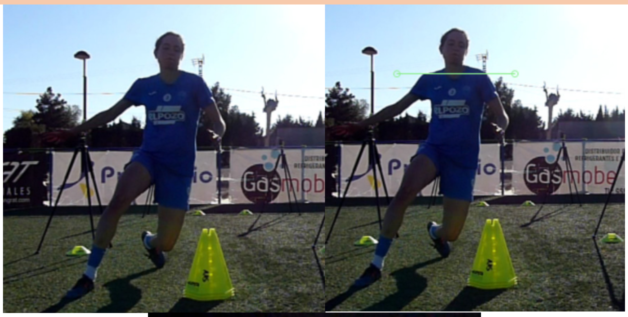

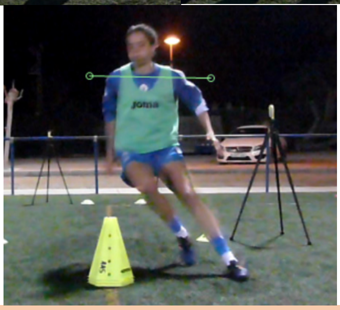

TR=2

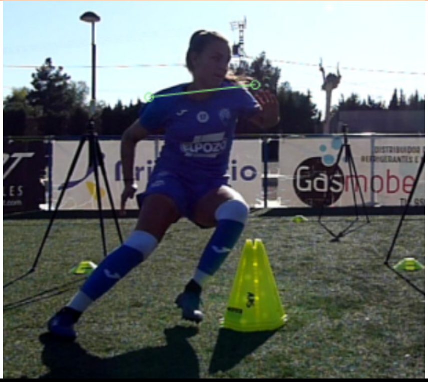

M=0

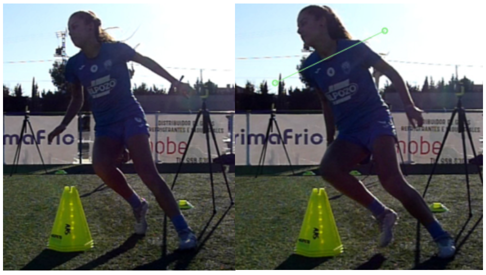

Sagital	**Tronco hacia atrás o vertical a lo largo del contacto final** (a lo largo de la fase WA)	**Nota explicativa**

MANUAL ANÁLISIS "CUTTING MOVEMENT ASSESSMENT SCORE (CMAS)" EN COD 90º (side-step)	
• Si el tronco permanece inclinado hacia atrás o vertical durante el contacto final, quiere decir que el desplazamiento del tronco en flexión no es adecuado	Para puntuar "No" debe observarse claramente que el tronco se desplaza hacia adelante a lo largo del FFC (**situándose el COM sobre el COP**).
Sí=1	No=0

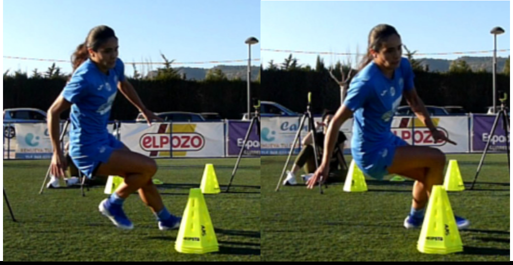

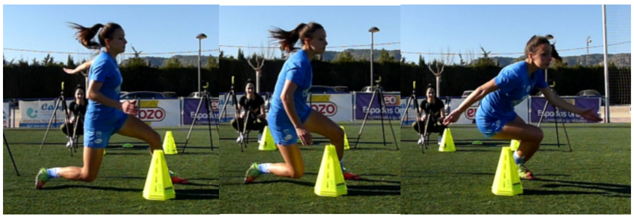

**Sagital**	**Flexión limitada de la rodilla durante el contacto final** (a lo largo de la fase WA)	**Nota explicativa**
	• Flexión de rodilla $\leq$ 30º (rigidez-no amortigua carga)	Para puntuar "No" debe observarse una clara flexión de rodilla (de la pierna de apoyo) a lo largo del FFC
	Sí=1	No=0

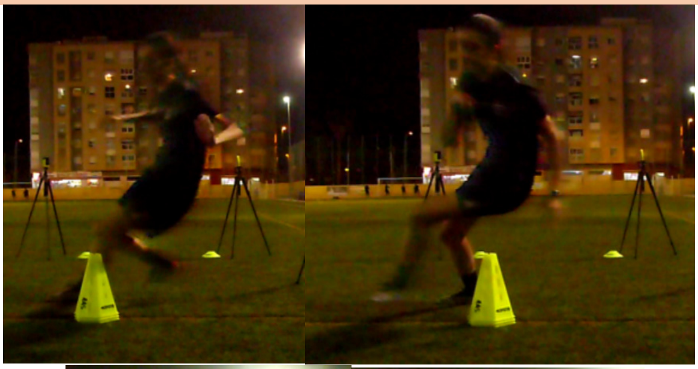
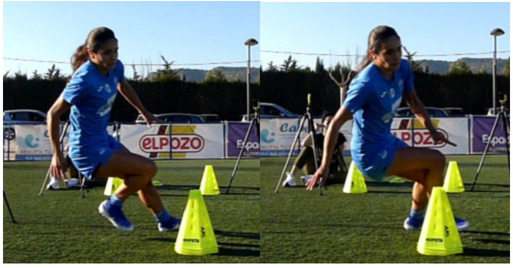

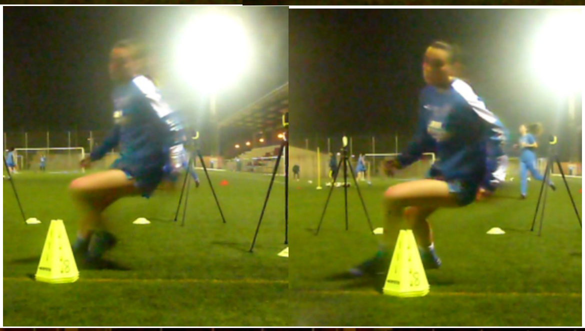
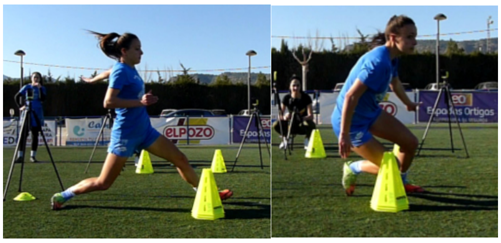

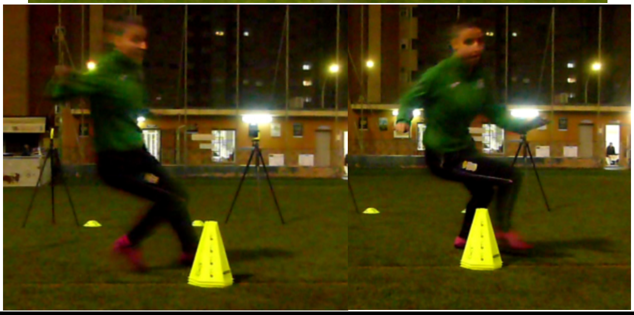
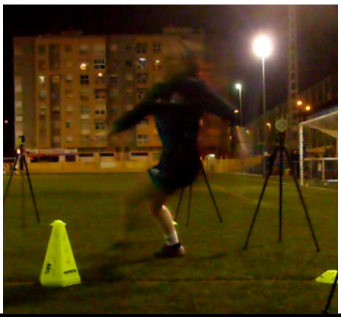

**Frontal**	**Excesivo movimiento de la rodilla en valgo durante el contacto final** (a lo largo de la fase WA)	**Nota explicativa**
	• Desplazamiento medial de la rodilla a lo largo del FFC	Para puntuar "Sí" debe observarse un claro desplazamiento de la rodilla en valgo durante el FFC. En caso de duda, podemos trazar el FPPA al inicio y cuando se observe el mayor valgo
	Sí=1	No=0

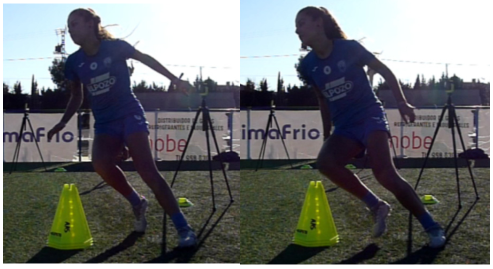
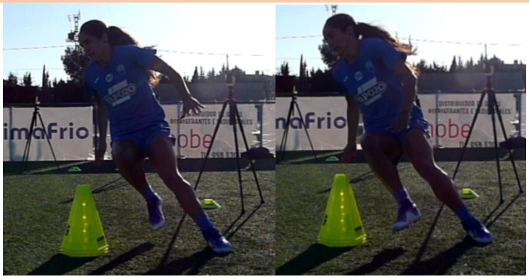

MANUAL ANÁLISIS "CUTTING MOVEMENT ASSESSMENT SCORE (CMAS)" EN COD 90º (side-step)

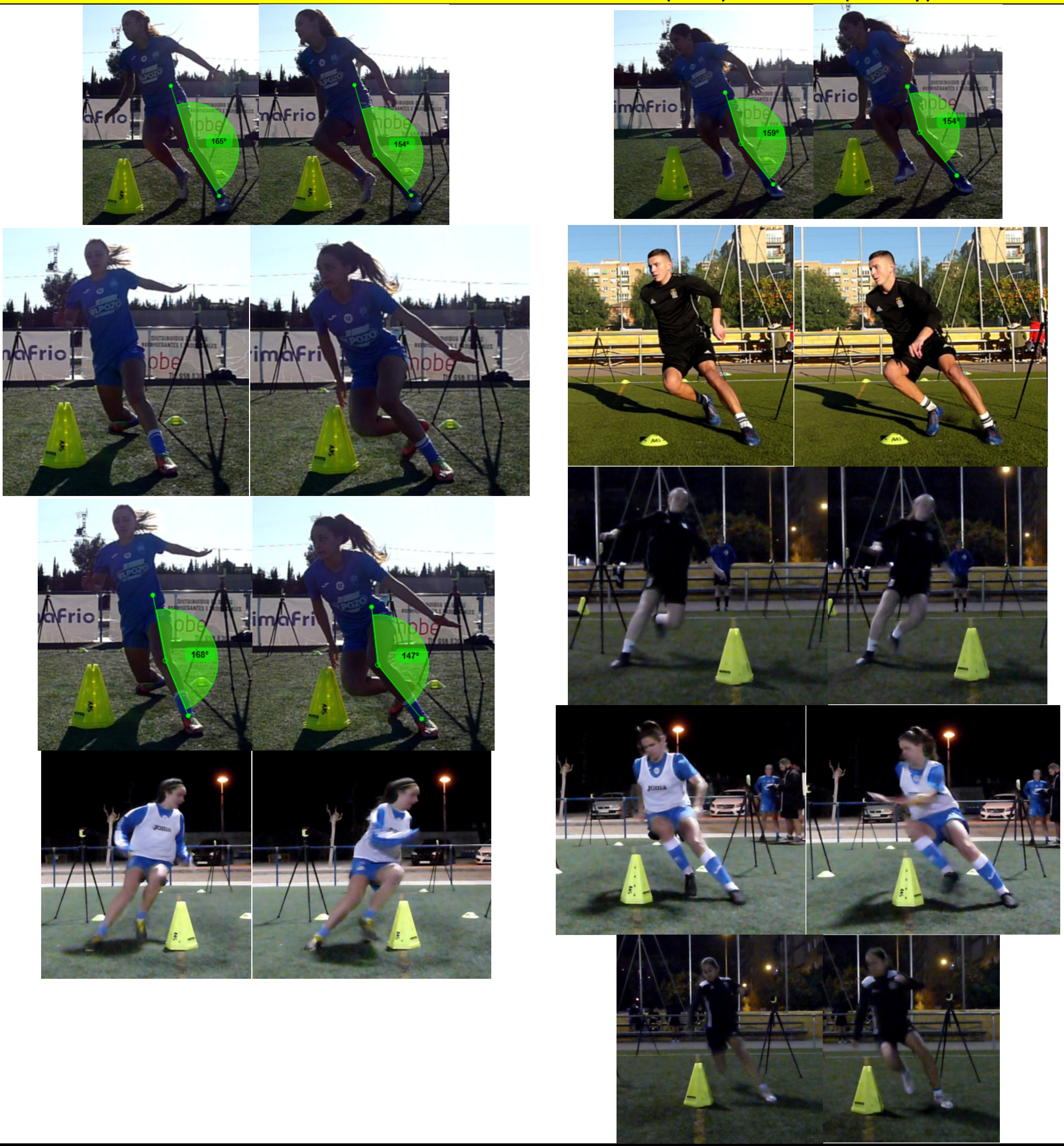

Total Score	/11

***Key = PFC: Penultimate foot contact; COM: Centre of mass; COP: Centre of pressure; WA: Weight acceptance phase; TR: Trunk rotation; Y: Yes; N: No; L: Lateral; U: Upright; M: Medial.**

La visualización de los vídeos se llevará a cabo a través de kinovea (versión 8.15). Se recomienda la visualización de la vista frontal y de la vista sagital a la vez. Los vídeos pueden visualizarse tantas veces como se requiera. Si existe duda, se aconseja optar siempre por la opción más conservadora.

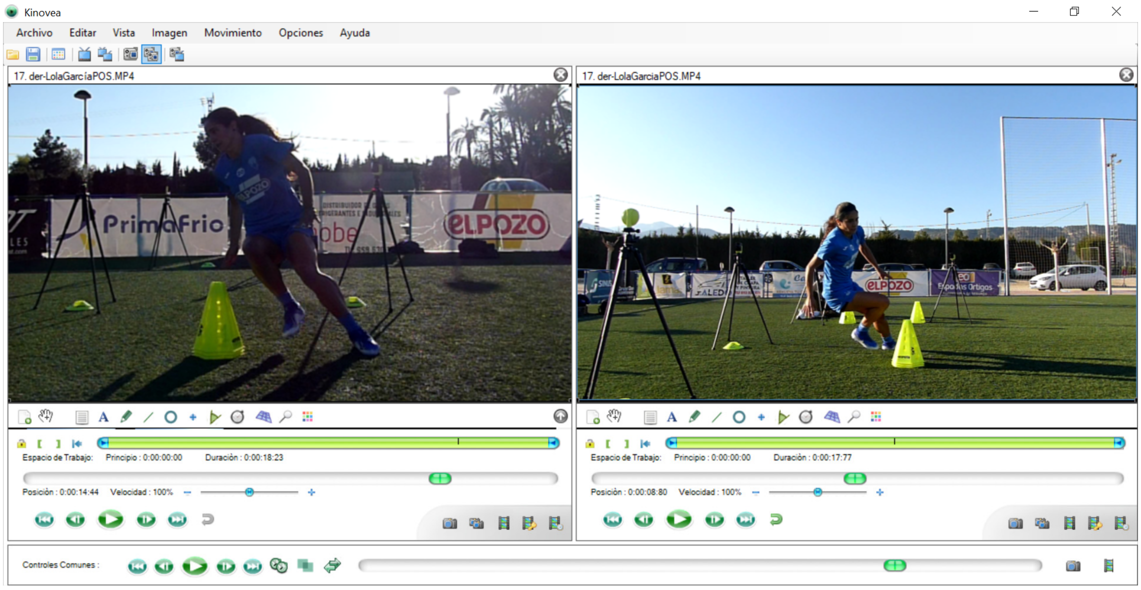

Supplement: Supplementary file 1 [file ijerph-19-04143-s001.zip › ijerph-1639560-supplementary.pdf]
